# Doctors as the governing body of the Kurdish health system: exploring upward and downward accountability among physicians and its influence on the adoption of coping behaviours

**DOI:** 10.1186/s12960-015-0039-x

**Published:** 2015-06-04

**Authors:** Goshan Karadaghi, Chris Willott

**Affiliations:** Department of Family and Community Medicine, University of Sulaimaniyah, Sulaimaniyah, Kurdistan Region Iraq; Institute for Global Health, University College London, 30 Guilford Street, London, WC1N 1EH UK

**Keywords:** Health system, Kurdistan, Discretion, Street-level bureaucracy, Discontent, Coping strategies

## Abstract

**Background:**

The health system of Iraqi Kurdistan is severely understudied, particularly with regard to patient-physician interactions and their effects. We examine patterns of behaviour among physicians in Kurdistan, the justifications given and possible enabling factors, with a view to understanding accountability both from above and below.

**Methods:**

An ethnographic study was conducted in the Sulaimaniyah Teaching Hospital in the Kurdistan Region of Iraq. Data was collected through negotiated interactive observation, and interviews were conducted with 10 participants, 5 physicians and 5 patients. Data collected was analysed using thematic analysis.

**Results:**

Common patterns of practice among physicians in Kurdistan include displays of discontent, reluctance to negotiate decisions with patients and unfavourable behaviours including dual practice and predatory behaviours towards patients. These behaviours are justified as a mechanism of dealing with negative aspects of their work, including overcrowding, low salaries and social pressure to live up to socially conceived ideas of a physician’s identity.

**Conclusions:**

Michael Lipsky’s theory of street-level bureaucrats and their coping behaviours is a useful way to analyse the Kurdish health system. Physician behaviours are enabled by a number of factors that work to enhance physician discretion through lowering of upward and downward accountability. Physicians are under very little pressure to change their behaviour, and as a result, they effectively become the street-level governing body of the Kurdish health system.

## Background

Health systems in the Kurdistan Region are largely unstudied. What little literature there is on the Kurdistan Region of Iraq (KRI) health service is highly critical of it [1–3]. Tawfik-Shukor et al. [3] describe the Kurdish health system as “centralised, politicised, nontransparent, disorganised with no clear governance, regulatory financing or accountability framework let alone vision or goals”. There are also accusations of corruption, predatory behaviour and mismanagement [1, 3, 4]. It is important to understand the roles and behaviours of physicians within the Kurdish health system in order to determine how patients experience it. Only if we understand patient experiences can we understand the quality of services provided by the system [5].

This article examines the health system of the KRI using Michael Lipsky’s [5] theory of street-level bureaucracy. We argue that examining health policy in the KRI is an inadequate method of understanding the way that citizens actually receive healthcare. In order to do so, one must understand the activities, behaviours and routines of doctors, nurses and other staff working in the KRI health system, conceptualized here as street-level bureaucrats. In this article, we focus on doctors and argue that the coping strategies they use in the course of their work directly influence the experiences of patients and that the current operation of the Kurdish health system leads to significant discontent on the part of both service providers and users.

### Theoretical framework

This article draws heavily on the theory of street-level bureaucracy elucidated by Michael Lipsky [5]. In his seminal contribution, Lipsky describes employees of the state with the discretion to dispense public rewards and sanctions, including teachers, law enforcers and health workers, as street-level bureaucrats (SLBs). According to Lipsky, SLBs have huge uncertainties within their work environment, the “defining facet for which is that they must deal with clients’ personal reactions to their decisions” [5]. Lipsky states that the behaviours of SLBs should be considered after understanding the contextual conditions under which they work. Negative pressures as well as constant exposure to dilemmas, for instance, the ever-present gap between demand for services and limited resources, as well as high workload and low government salaries [5–8], are said to compel SLBs into behaviours and practices that enable them to cope with such unfavourable conditions. Macq et al. [8] state that the low government salaries, combined with the high expectations SLBs have of their work, mean it is inevitable that SLBs will “seize opportunities that are rewarding professionally and financially” [8].

A factor that enables the development of coping strategies is the considerable discretion afforded to SLBs, which exists primarily because without it, the state would become slow and unresponsive. For example, a health professional’s discretion in decision-making is essential to the effective management of highly variable clients and situations, which may often be emergency cases [6]. Blundo [9] explains that within Senegal a weak sense of accountability to the public is another enabling factor that allows the “daily negotiation” of resource allocation, access to services and the application of policy to suit the provider rather than the user of services. Lipsky [5] argues that, “the decisions of SLBs, the routines they establish, [and] the devices they invent to cope with uncertainties and work pressure effectively become the public policies they carry out.”

Understanding the behaviours and practices of SLBs as well as the reasons behind them enables an understanding of policy and its application. This has been described as “real governance” by Olivier de Sardan [10]. Although the concept of street-level bureaucracy is widely accepted, it has not been extensively studied within the health sector. In fact, there is limited empirical evidence that health professionals resort to coping strategies [11]. Therefore, the exploration of the theory of street-level bureaucracy in the health sector, as well as medical staff and their coping mechanisms, is a significant gap within the literature.

A further important reason for the discussion of SLBs in health lies in the asymmetry of information that exists in healthcare. This means that health workers, and physicians in particular, have a great deal of power over their patients precisely because of this information gap. How they use this power has a critical impact on the way that resources are distributed in the healthcare sector.

### Setting

Kurdistan is a vast region in the Middle East, including part of four different countries: Iraq, Iran, Syria and Turkey. The KRI is currently the only region with a semi-autonomous government and has been formally recognized as Kurdistan. There have been moves towards independence for Iraqi Kurdistan in recent months.

This region has undergone consecutive wars, internal conflicts and international and domestic sanctions over the last four decades. This resulted in the demise of the health system from one of the best in the Middle East in the 1980s to an out-dated, heavily overcrowded and inefficient system today [3–12]. The isolation of the area during the 1990 sanctions and Gulf War meant that Iraq’s health professionals were cut off from the world, resulting in a generation of physicians who graduated with inadequate training and poor motivation [1, 13]. Along with being poorly motivated, physicians in Kurdistan have also been described as patient-unfriendly, corrupt and inefficient [1–3, 7]. There is low expenditure on health services in Iraq – 4.1 % of GDP in 2008 [1] – which combined with an overcrowded health system gives rise to undesirable working conditions among health professional in KRI hospitals.

## Methods

An ethnographic methodology was used to conduct this study. Data was collected by Goshan Karadaghi (GK) from April to June 2013 within the Sulaimaniyah Teaching Hospital, situated in Sulaimaniyah, the second largest city of the KRI region. Data was collected within the consultancy ward and the surgical emergency ward of the teaching hospital. These two wards are very different. The consultancy ward consists of a number of examination rooms wherein specialized doctors are seated and patients queue to see them. However, the surgical emergency ward is mainly covered by junior or less experienced doctors, who are supervised by specialized physicians. In the surgical emergency ward, physicians are expected to go to patients. These two settings gave the opportunity to observe physician behaviour within structurally different work conditions.

During data collection, GK conducted what Wind [14] terms “negotiated interactive observation”, where she sought to retain her role as researcher rather than seeking to take on the role of physician, patient or visitor. This method is said to “capture what happens when doing fieldwork without at the same time assuming that you become one of [the participants]” [14]. This enabled GK to gain greater access to information from both patients and physicians without compromising ethical guidelines.

During observations, informal data was collected including observations of physician behaviour, patient behaviour, details of physician-patient interactions and informal conversations with participants regarding the subject of study. All participants were Kurdish, as GK’s inability to speak or understand Arabic, the other language spoken in KRI, was a barrier in approaching such individuals. Ten participants were interviewed, five physicians and five patients. Physicians who were interviewed had been approached during observations, while patients were approached outside of hospital settings. The interviews were done in public places, where the participants felt comfortable, enabling participants to speak freely, away from the gaze of people involved in health services, as well as upholding the anonymity of the participant. Interviews were voice recorded and digitally transcribed.

Sampling was done through a combination of convenience and snowball sampling. Initial contacts with physicians were made through a gatekeeper, and further contacts were made through these physicians. Patients were recruited using a convenience sampling technique.

A thematic analysis framework was used to analyse the data. This is an inductive process of analysis, wherein hypotheses and themes emerged from the data itself. Long table analysis methods were used during the continuous cycles of familiarizing, coding and thematic categorization with every new addition to the data set. As a result, a number of themes and subthemes emerged from the data.

GK is a Kurdish female who has been brought up in the United Kingdom, with a mix of both Kurdish and British cultural values. This means that her outlook and the way she understands cultural cues are through exposure to both British and Kurdish culture. This aspect of her identity meant that her awareness of Kurdish culture and social protocol was perhaps more than that of a non-Kurd in the field. This was as much a hindrance as it was a help, as of course knowing a culture enables greater integration particularly if you are considered “one of them”. However, as she is considered a member of the Kurdish community and answers to those social rules, this meant that she was not as free as other researchers would be. For example, as an unmarried woman, she could not approach male participants without being introduced to them first, as to do so would have been against social protocol and would have had an effect upon her reputation within the community. Communication with male participants in particular had to remain to a certain extent formal for the same reason.

We do not believe that GK’s presence had much of an effect on the data gathered. In the initial period of data collection, physicians may have felt under scrutiny and behaved differently from normal, but as time passed, they became much more at ease. It is also the case that patients in KRI hospitals are often seen two or three at a time, so both doctors and patients are used to “others” being present during consultations.

There are some limitations to the methodology adopted, most particularly that research only took place in one hospital, so generalizing beyond this one case is difficult. This is often a problem with ethnography. Nonetheless, we argue that our findings chime with other research in this area and can therefore be seen as an important addition to the existing literature on the topic of the everyday operation of health systems in the Middle East.

### Ethics

Ethical approval for conducting this research was granted by the UCL ethics committee and by the Sulaimaniyah directorate of health and the Ministry of Health of the Kurdistan Regional government. All observed and interviewed participants were informed of the nature of the study, the exact nature of their participation and the possible uses of the study through an information sheet. After it was established that participants understood the aims and intentions of the study, they were asked to sign consent forms. Special ethical consideration was given to truthfulness, confidentiality and security of participants [15]. All patients were approached outside of the hospital and the gaze of physicians, to avoid the possibility of physicians identifying participating patients in case they change the way they act towards them.

## Results and discussion

### Discontent

Throughout observations of patient-physician interactions within the Sulaimaniyah Teaching Hospital, a recurring pattern of behaviour that physicians demonstrated was that of displays of discontent. Physicians openly express emotions like anger, frustration and impatience regularly when interacting with patients, through both physical manners – the use of dismissive body language and facial expressions – and verbal manners – raising one’s voice, speaking down to patients and the use of implicit language suggesting contempt. Displays of discontent are not only expressed by physicians, however. Medical assistants and porters also display impatience and frustration. It is a practice that is a common feature of the KRI health system and forms quite a hostile environment for patients.

Physicians in Kurdistan justify these patterns of behaviour most commonly as a means of dealing with an over-utilized system. The Kurdish health system is intensely overcrowded [1, 2, 4]. The hospital-based health-seeking culture, lack of appointment system and the fact that the public system is free, or has a very minimal charge for services, mean that a large number of patients utilize the services available. Physicians identify the conflict between limited resources and high demand that arises as a result of these conditions [5, 6, 8]. However, in this context, the resource that is demanded most is the time and attention of physicians. One physician explained that doctors may see up to 200 patients within a 6-h shift.

These unfavourable working conditions may be a justification for the adoption of coping behaviours, as described by Lipsky [5]. The overloaded system, along with patient expectations of being seen quickly, encourages physicians to adopt behaviours that enable the quick processing of patients and the prevention of burnout [16].

The expression of anger and impatience, especially if it is not retaliated against, can also be viewed as a method of establishing one’s authority [16]. Within the strongly hierarchical Kurdish society, physicians hold a senior social position and are described as an elite social group both by physicians and patients. Physicians are granted a position of prestige, primarily because of the perceived “sacred” and positive nature of their work and the fact that they historically have been the wealthiest and most educated members of society [12]. This grants them a respect that all members of society, particularly patients, are obliged to show. Signs of submission, through lowering one’s head or placing one’s hand on one’s chest, are acknowledgements of status of people of social importance. These behaviours are brought into the consultation room and therefore shape the manner with which patients and physicians interact. To remind patients of their authority, through the display of discontent, physicians are in effect reminding patients of their relative inferiority and the value of the physician’s time as a resource. As one patient explained:

You have to respect doctors, you have to basaqa and baqurban (plead with) them…because they might get angry and make your treatment more difficult.

This may play a part in explaining the common submissive nature of patients that GK observed during her fieldwork and more significantly explains the lack of resistance that patients showed towards physicians and their decisions. Nielsen [7] found that SLBs respond more positively to patients who are obliging and respectful, because treating such individuals is less complicated than treating hostile patients. Some physicians did express their preference for compliant patients, particularly as this means that physicians have a greater discretionary freedom in decision-making. We argue that considering the demand on the physician’s time, these displays of discontent act to deter patient resistance and input into the decision-making process in order to limit the time required for each consultation, as well as to smoothen the physician’s journey through the working day. This form of behaviour is enabled because of social principles of respect and status. The social protocol of respecting hierarchically superior individuals such as physicians is to acknowledge their authority and therefore to increase their discretionary freedom [17]. This discretionary freedom is a significant feature of SLB theory [5], as it is an enabler of coping behaviour adoption.

### Lack of health awareness

The lack of health awareness among patients is a factor that is widely identified by physicians as a problem that adds to their workload, compelling physicians to adopt coping strategies, and therefore is used to justify displays of discontent. It is also used as a justification for another common practice: the reluctance to explain conditions, their causes or their management to patients. This reluctance is justified as a means of dealing with limited time. Within the 2- to 3-min time slot designated for each consultation [3], it is impossible to expect in-depth explanation or negotiation of decisions, a fact which is exacerbated by the low level of health knowledge among patients in Kurdistan. Physicians explain that even if time constraints were not an issue, still the “reassurance of patients is futile, patients don’t understand, they are incompetent”:

I don’t know, I try to explain, but sometimes if you do its not understood by the patient, I don’t really have 30 minutes to spare for every patient to explain a procedure especially if they still don’t understand… so most doctors don’t, most prefer to just say ‘you need an operation, come in on Tuesday’.

We would argue, however, that there may be another motive behind the lack of explanation of decisions regarding a patient’s health and that is that maintaining an asymmetry of knowledge allows the physician to maintain power over the patient [18, 19]. This acts to enhance one’s discretion, therefore limiting resistance to one’s authority and increasing the respect and compliance of the patient. Nielsen [7] argues that the greater the level of knowledge of patients, the greater their negotiating ability. Although the logistics of communicating health knowledge is the justification used by physicians to explain the lack of explanation given to patients, one can see how the lack of sharing of knowledge acts to exacerbate unequal physician-patient power relations. Patient-physician interactions demonstrate the distance between physician and patient not only in knowledge but also in status [17]. Physicians described themselves as an elite social group that rarely mixes with general society. This social separation is often attributed to the high position of physicians in the social hierarchy and the respect that is attributed to such a position [20, 21]. Patients noted their resentment of this separation to GK during the research.

However, any demonstration of discontent from patients towards physicians within interactions was not observed. This is likely to be due to respect and status. Physicians appear to assert their status through behaviours that separate them from others, for instance, how they dress, or speak, or indeed where they choose to sit in the wards – often behind a large desk, quite distant from the patient. We acknowledge that behaviours that encourage physical separation may be assimilated, as the culture within which physicians are formed instil these behaviours as normal. However, such behaviours, whether intentional or assimilated, remind patients to give physicians the respect society grants them. One physician described how social expectations shape such perceptions:

I remember in medical school, one of our teachers comes in and says, you are above the rest you should act like you’re above the rest, you are the leaders of the leaders, you are the top of the top so act like this, behave yourselves.

This social separation encourages the perception of patients and physicians being on opposing sides. A doctor claimed that patients “consider us [physicians] as enemies”.

### Unfavourable behaviours

The social separation and status of physicians is a key determinant of power relations. However, with regard to unfavourable behaviours, it is the social expectations of physicians that are of great significance. By unfavourable behaviour we refer to acts of physicians that would normally be deemed to have a negative impact on the services that are provided for a patient or the health service in general. One example of this is participation in dual practice. This is a common feature of the Kurdish health system, wherein the majority of physicians work in both public and private sectors. Macq et al. [8] describe that physicians in dual practice often just compete with themselves and therefore conflicts of interest arise as a result of this [2, 22]. This point is used to explain the observed – sometimes intentional – demise of public sector quality of care. These observations have been acknowledged by physicians in Kurdistan also, wherein two physicians argued that motivation for work within the public sector is considerably less than in private practices. Physicians justify working in the private sector as it allows the standard of living that is closer to what physicians expect. A doctor explained that public sector salaries are not enough to live the lifestyle that both physicians and society expect physicians to live:

Doctors get $1200 [per month] for their work when they start. You become settled as a doctor at 34+ years. Iraqi law dictates that you retire at 63 - within this time you need to achieve what society expects doctors to be…. doctors are surrounded by other doctors with a nice car, a few flats and a plot of land for weekends. You cannot achieve this on a government salary.

Ferrinho and Leberghe [11] claim that a way to explain these practices is to understand that health professionals are often socialized within a context where they themselves and society come to expect a standard of living that cannot be met by government salaries. This description is quite fitting with regard to the KRI. Physicians regularly referred to the pressure they felt from social expectations of them. Hegemonic groups of society, in this case physicians in Kurdistan, are characterized by their disproportionate possession of what Sidanius and Pratto [23] call “positive social value” referring to material and symbolic things that people aspire to having. Physicians are, within Kurdish society, a social group that children are encouraged to aspire to. A patient explained that this is because of the monetary security that is assumed to arise from being in the medical profession. A doctor described the disparity between what society expects physicians to have and what they can afford on government wages, and another referred to the judgement that physicians face based on their material expression of wealth and therefore status:

A simple thing like taking the bus, they say ‘look at that doctor, he is so cheap’. They lose respect for you because they laugh at you, so you can’t accept that, and you buy a car, but even that must be a flashy new one, otherwise ‘what sort of doctor are you?’

Hence, to have the “nice car, a few flats, and a plot of land for weekends” is, in the mind of many physicians, a factor that enables them to uphold social expectations and, more importantly, the respect of society. Therefore, it is justified for a physician to pursue extra income outside the public sector. These motives of moonlighting for income have been exacerbated by the recent post-war economic boost that the KRI has enjoyed [24]. This economic boost has meant that pre-existing social hierarchy and patterns of education, wealth and status have changed. There is resentment from physicians towards individuals who are less educated and conventionally are of a lower social status but who have the financial ability to compete with physicians:

A doctor looks and sees an uneducated man who doesn’t even know how to sign a document is a contractor and is a millionaire. But all the doctors put together won’t have as much as one contractor. This causes animosity among people.

Therefore, physicians feel the need to compete with such individuals in order to uphold their positions of status within Kurdish society. This demonstrates the importance of material indicators of wealth and the attributed status that is granted with such expressions of wealth within Kurdish society [24]. Along with dual practice, however, physicians have been accused of predatory behaviours within public health services for private service gain. Although such behaviours were not witnessed during observations (perhaps as a consequence of GK’s presence), physicians and patients commonly mentioned such behaviours among physicians. These accusations include pressuring patients from public services into private clinics and the use of public hospital resources for private patients. One doctor stated:

Yes the doctor uses the public sector for his private gain. He admits patients from his private clinic, uses government resources for their operation and sees the patient in the private clinic after.

These behaviours have also been documented in other studies of the Kurdish health system. Tawfik-Shukor et al. [3], for instance, describe physicians in Kurdistan as having a “parasitic orientation towards the use of public hospitals”. They argue that physicians in the public sector have little incentive to promote public health services, because of low pay and being overworked. Therefore, they pursue job satisfaction by increasing their income through working in the private sector. We argue, however, that in this instance Lipsky’s definition of SLBs being compelled to adopting behaviours because of the negative aspects of their work – in this case, social judgement – may be too simplistic an explanation. Nielsen [7] describes the possibility that SLBs may be equally enticed into coping behaviours in order to reinforce or seek out positive rewards. Physicians may be compelled into pursuing extra monetary income to avoid social judgement, but the sense of accomplishment they get from being able to afford items of positive social value may also be a significant driving force in the maintenance of such behaviours. Similarly, with the increase in wealth comes a reinforced social status and social respect, much like the outcome of displays of discontent and reluctance to negotiate information. Physicians may feel they have to undertake such coping behaviours to deal with a heavily overcrowded system, and to avoid social judgement, but we argue they may also want to behave in such ways because such behaviours are reinforced by the positive benefits they may obtain.

Patients attribute the pursuit of such behaviours to physicians being “money hungry”. Physicians deny this and explain that the money they generate is to satisfy social expectations, as well as their personal expectations of what a physician’s lifestyle is supposed to be. By doing so, they uphold their social standing. The negative perception of the behaviours of physicians by patients, however, does not influence their behaviour. SLBs are technically accountable to their public [5, 25], though in this instance, although there is apparent discontent among patients, it has little influence on the behaviours of physicians.

### Discretion and accountability

Behaviours such as displays of discontent, limited communication of information and unfavourable behaviours are justified as ways of managing negative pressures of physicians’ working conditions. There are a number of enabling factors that allow these behaviours to be adopted by limiting accountability and consequently increasing the discretion of physicians, the first of these being a general presence of fear and lack of trust towards the Kurdish health system and its physicians.

Trust among patients towards physicians is low, evident in the common practice of patients seeing multiple physicians for one condition, to check the accuracy of diagnoses and treatments. Physicians acknowledged this lack of trust and primarily attributed it to the role of the media in exposing scare stories about the system and the lack of education of patients for believing them. Patients mention these stories as a justification for their mistrust of the competence of physicians in Kurdistan.

The intentions of physicians are also questioned by patients, a number of whom brought up the maternity ward as an example to demonstrate this. There is a belief that patients are denied caesarean sections within the maternity wards because it gives physicians an opportunity to force people into their private clinics for the procedure. Physicians do not deny that they take advantage of the situation. One doctor explained that a history of using physicians as scapegoats for the inadequacies of the health system both under the Ba’ath regime and the current regional government have only fed these feelings of mistrust and fear among patients.

Another possible factor, which adds to the mistrust and fear of physicians, is the recurring practice of doctors dismissing diagnoses and treatments that other physicians have identified for patients. Similarly, they openly criticize the competence of other physicians in front of patients. This behaviour may be explained by the competition that arises when physicians work within the private sector, as to berate a fellow physician is to limit the competition for your clinic, especially as patients choose to see private physicians based on word of mouth and the reputation of the doctor among the community. This common mistrust is often used as a justification of the fear that patients feel towards physicians, along with their anticipation of being shouted at or dismissed. This fear acts as an enabler of adopted behaviours, because it exacerbates the inequality of the physician-patient power relationship. To fear a physician is to grant them greater power over interactions and, therefore, grant them greater discretion in decision-making. This discretion can also be enhanced through the limiting of accountability that physicians have both in interactions with patients and with regard to top-down regulation.

SLBs are characteristically held accountable through both bottom-up and top-down regulation [26]. However, within the Kurdish health system, physicians have very little accountability to patients, primarily due to physician-patient power relations. The hierarchical separation of physicians and patients and the submissive identity that patients are granted mean that they are denied their ability to hold physicians accountable. This fact is exacerbated by the fact that the paternalistic nature of interactions means that physicians control resource access and allocation. Skirbekk et al. [26] state that “[p]atients are, whether they like it or not, whether they think of it or not – at risk regarding the competence and goodwill of the physicians”. A patient expressed this unmistakeably when she describes that:

You can’t really tell a doctor, what you’re doing is wrong, because even if you do, nothing will change, except that the doctor will probably dismiss you, and you don’t get treated.

There is a perception among patients that even if attempts were made to address these behaviours, as a patient said, “nothing will change”. There is a belief that physicians are untouchable because of the concept of “psht” or backing.

Many of those doctors have ‘psht’, they have friends in high places with political power, so even if you were to complain nothing would happen, a few wastas [social favours] here and there would make sure of that.

Therefore, what patient protective policy is present or is established in the future may not be effective if patients and physicians themselves feel physicians are above the law.

Physicians have very little if any top-down accountability either. There is no monitoring of physician activity or regulation of private-public health sector relations [1–3], and physicians know this. One physician referred to the prevalence of social relationships within Kurdish society. Many physicians have powerful connections with policy makers. These relationships, the fact that the KRI region still suffers from a shortage of physicians and the sheer number of physicians participating in these behaviours mean that the regional government cannot do much to regulate physicians using top-down approaches either.

This point is reiterated by Miller [27] who states when describing corrupt behaviours in Eastern Europe that it is “not so much the morally corrupt few, as the behaviourally corruptible many” who are the problem. Blundo [9] explains the influence these conditions have on top-down regulation when he argues that in certain circumstances where the state has no real solution to deal with behaviours of SLBs, it voluntarily averts its gaze and by default forms a permissive space that consequentially legitimizes such irregular practices. In essence, because patients aren’t given the assurance that physicians will be reprimanded, they withhold their complaints and comply with the whims of physicians, as they in effect are the governing body of the Kurdish health system. Therefore, the lack of top-down regulation deters the presence of street-level regulation.

As a result of these factors, physicians within the Kurdish health system have considerable discretion regarding their practices and control of resources. This is a significant factor that enables the adoption of coping behaviours, because it not only limits the obstacles to them but also by default legitimizes these behaviours. Physicians therefore are in a state of absolute discretion in behaviour and consequentially in the making of street-level policies. Therefore, policy change may be a futile means of addressing these issues if the system of accountability is not addressed.

## Conclusions

This article has opened a window into the Kurdish health system and how the wider cultural context influences decisions, interactions and behaviours within hospitals. Behaviours within interactions such as displays of discontent and the reluctance of physicians to share health information, as well as unfavourable behaviours such as dual practice and predatory behaviours towards patients and the health system, have been identified within Kurdish hospitals as common patterns of practice. One may label these behaviours as forms of coping behaviours as Lipsky would describe, as they are justified as mechanisms of coping with or making the best of highly negative conditions of work. These negative conditions are attributed to high workload, low salaries and conflicts between demand and access to resources [5–8] although positive influences appear to play a role in enticing physicians into these behaviours also. However, the most significant finding of this study was the identification of factors that enable these coping behaviours. Power relations play a significant part in this and are fuelled by society’s hierarchical values, the distance between physicians and patients – both because of social status and information asymmetry – and the prevalence of both fear and lack of trust towards physicians. These power relations reinforce physician authority and patient inferiority. This limits the power with which patients may hold physicians accountable, and therefore, there is limited if any bottom-up regulation of physician behaviour.

The desperate need in the region for physicians, as well as the widespread culture of these adopted behaviours and the lack of top-down regulatory bodies, leaves a health system that is *de facto* governed by physicians. This is an absolute condition of discretionary freedom, where physicians are free to do as they please, and this is the defining factor that explains the adoption of behaviours that are common practice among doctors in Kurdistan.

## Abbreviations

KRI: Kurdistan Region of Iraq
SLB: street-level bureaucrat
